# In Memoriam: Professor Enio Buffolo (1941 - 2025)

**DOI:** 10.21470/1678-9741-2025-0409

**Published:** 2026-02-18

**Authors:** Ricardo de Carvalho Lima, José Wanderley Neto, José Teles de Mendonça, Paulo Roberto Brofman, Fernando Antonio Lucchese, Fabio B. Jatene, José Medina Pestana, Tomas Salerno

**Affiliations:** 1 Universidade de Pernambuco - UPE, Recife, Pernambuco, Brazil; 2 Universidade Federal de Alagoas - UFAL, Maceió, Alagoas, Brazil; 3 Universidade Federal de Sergipe - UFSE, Aracajú, Sergipe, Brazil; 4 Pontifícia Universidade Católica do Paraná - PUCPR, Curitiba, Paraná, Brazil; 5 Universidade Federal do Rio Grande do Sul - UFRGS, Porto Alegre, Rio Grande do Sul, Brazil; 6 Universidade de São Paulo - USP, São Paulo, São Paulo, Brazil; 7 Escola Paulista de Medicina, Universidade Federal de São Paulo - UNIFESP, São Paulo, São Paulo, Brazil; 8 Miller School of Medicine, University of Miami, Miami, Florida, United States of America

**Keywords:** Cardiovascular Surgery, Enio Buffolo, OPCAB, TAVI, Brazil, Surgical Innovation.

## Abstract

Professor Enio Buffolo, born on Dezember 9^th^, 1941, was a pioneer in thoracic and cardiovascular surgery in Brazil. His career spanned over six decades, during which he made groundbreaking contributions to world cardiac surgery, including the development of off-pump coronary artery bypass, endovascular treatment of aortic aneurysms, and the introduction of transcatheter aortic valve implantation in Brazil. He held numerous leadership roles, mentored generations of surgeons, and published extensively. His legacy is defined by visionary leadership, academic excellence, and deep humanistic values that continue to shape cardiovascular medicine in Brazil and beyond. If unanimous opinions are rare, just as rare are those that serve as examples, in their most diverse conducts, for family, friends, and coworkers. Dr. Enio Buffolo has a special place among this category of citizenship and humanism.

## INTRODUCTION

**Figure:**
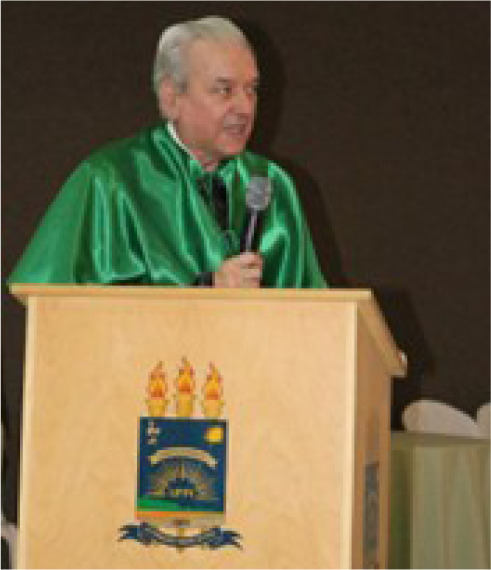
*Professor Enio Buffolo (1941 - 2025)*

**Table:** 

Abbreviations, Acronyms & Symbols	
ABCCV	= Academia Brasileira de Cirurgia Cardiovascular
EPM	= Escola Paulista de Medicina
GP	= Grand Prix
OPCAB	= Off-pump coronary artery bypass
UNIFESP	= Universidade Federal de São Paulo

Professor Enio Buffolo, a pioneer in thoracic and cardiovascular surgery in Brazil, passed away on October 6^th^, 2025, at the age of 83. He is remembered as a brilliant surgeon, visionary academic leader, and exemplary human being - steadfast in his commitment to knowledge, excellence, and service. His life is best captured by Bertolt Brecht’s timeless reflection:

“There are men who struggle for a day, and they are good. There are others who struggle for a year, and they are better. There are those who struggle for many years, and they are very good. But there are those who struggle all their lives - these are the indispensable ones.”

## EARLY LIFE AND EDUCATION

Born into a middle-class family in São Paulo, Brazil, Buffolo’s early years were marked by harmony and solidarity. After a vocational test suggested either history or medicine, he chose the latter and entered the Escola Paulista de Medicina (EPM) - now the Universidade Federal de São Paulo (UNIFESP) - in 1960. At EPM, he excelled academically and athletically, playing semi-professional indoor football and actively participating in the Athletic Association of the Medical School. Initially drawn to neurosurgery under the guidance of his uncle, neurologist Dr. Otavio Lemmi, Buffolo’s path shifted toward cardiac surgery through mentorship from Professors Aluísio de Matos Pimenta, Sergio Paladino, and Hugo Filipozzi - one of Latin America's pioneers in extracorporeal circulation.

## PROFESSIONAL CAREER AND LEADERSHIP

**Fig. 1:**
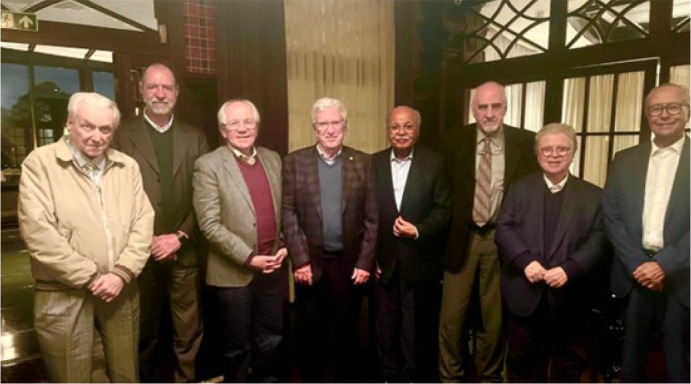
51^o^ Congresso Brasileiro de Cirurgia Cardiovascular, Curitiba, Paraná, Brazil, in July 2025. A memorable moment with Enio Buffolo surrounded by close colleagues and friends of the Sociedade Brasileira de Cirurgia Cardiovascular, celebrating decades of shared professional achievement and friendship. From left to right, Enio Buffolo, Fábio Jatene, Ricardo Lima, Paulo Broffman, José Teles, José Camargo, Fernando Luchesse, and José Wanderley Neto.

Professor Enio Buffolo graduated in 1965, having assisted in EPM’s first open-heart surgeries using extracorporeal circulation. His residency began in 1966, and he earned his PhD in 1973. Then in 1976, he completed his Post-doctoral Habilitation thesis at the Universidade de Mogi das Cruzes, São Paulo. By 1989, he was appointed Full Professor of Cardiovascular Surgery at EPM-UNIFESP and later served as Head of the Department of Surgery. Under Buffolo’s leadership, EPM-UNIFESP’s Department of Thoracic and Cardiovascular Surgery became a national reference center, performing over 46,000 procedures - averaging eight cardiac surgeries daily across multiple hospitals. Even after retiring in 2019, he continued teaching and operating at a private hospital Hospital do Coração in São Paulo. He held numerous leadership roles including President of the Sociedade Brasileira de Cirurgia Cardiovascular, Founding President of the Sociedade de Cirurgia Cardiovascular do Estado de São Paulo, Vice President of the Sociedade Brasileira de Cardiologia, and Founder and First President of the Academia Brasileira de Cirurgia Cardiovascular (ABCCV) (Figure 1).

**Fig. 2:**
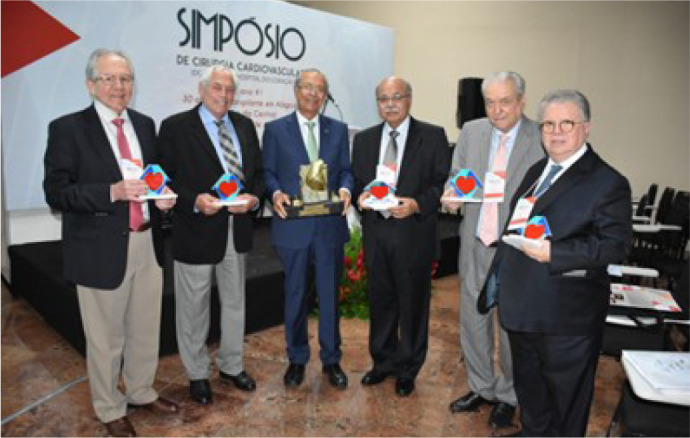
Founding of the Academia Brasileira de Cirurgia Cardiovascular, Maceió, Alagoas, Brazil (2021). From left to right, Ricardo Lima, Mozart Escobar, José Wanderley Neto, José Teles, Enio Buffolo, and Fernando Lucchese.

Together with a group of close surgical colleagues who shared and fulfilled their professional dreams over the course of five decades, Buffolo co-founded the ABCCV with the mission of uniting surgeons from all regions of Brazil and abroad under a common spirit of collegiality and institutional purpose. A historic photograph taken in Maceió, Alagoas, Brazil, captures the formal moment of the Academy’s founding. As its first president, Buffolo leaves an enduring legacy, preserved through his pioneering contributions to cardiac surgery and through the many disciples he trained and inspired (Figure 2).

## SCIENTIFIC CONTRIBUTIONS

**Fig. 3 A and B:**
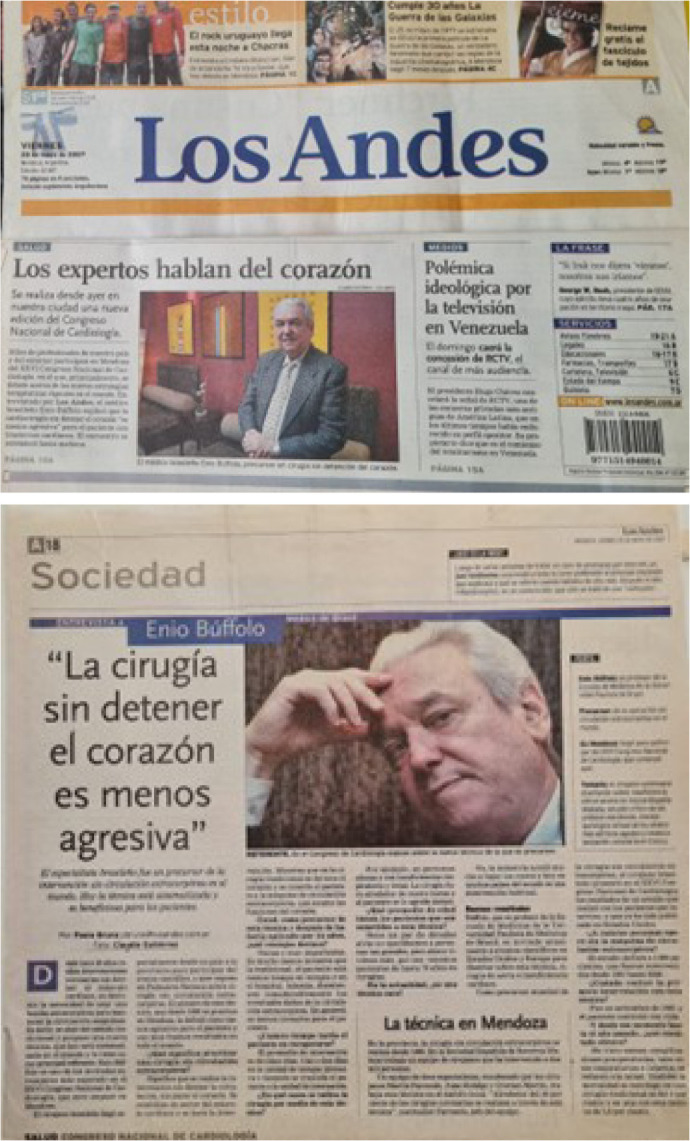
Myocardial revascularization surgery with a beating heart (off-pump coronary artery bypass) had worldwide repercussions on all continents.

Enio Buffolo and his surgical team were pioneers in the development and clinical adoption of off-pump coronary artery bypass (OPCAB) surgery in the late 1980s, profoundly influencing global cardiac surgical practice. After years of rigorous evaluation and debate, OPCAB was ultimately worldwide recognized as a safe, reproducible, and effective alternative to conventional coronary artery bypass grafting (Figure 3A and 3B). The OPCAB initial publication was in 1982 in the Brazilian Archives of Cardiology^[1,2]^.

Another major contribution was the introduction of the “elephant trunk” technique for the treatment of descending aortic aneurysms and type B aortic dissections in Brazil. This innovative approach simplifies complex aortic surgery and significantly improved outcomes, with notably low mortality rates. He subsequently led the development of endovascular stent graft technology in Brazil, with one of his early landmark procedures featured on the BBC's Tomorrow's World TV program^[3,4]^.

In collaboration with medical company Braile Biomedica, he also played a central role in introducing transcatheter aortic valve implantation in Brazil, helping to establish the first domestically manufactured prosthesis for use in the national public health system (Sistema Único de Saúde) and registering the first patent for this prosthesis in all Latin America^[5]^.

**Fig. 4:**
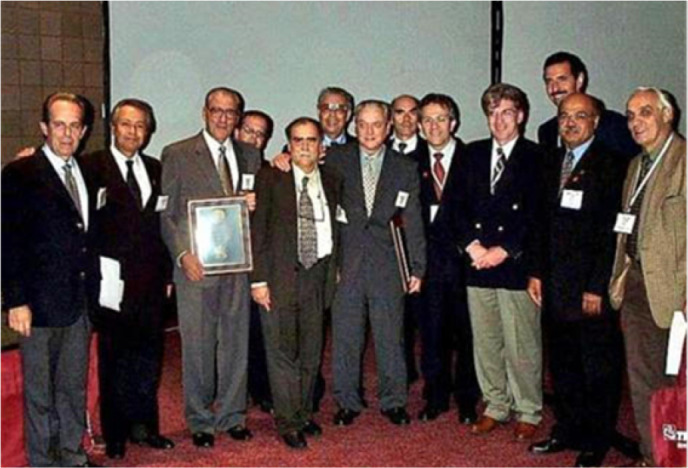
The Thirty-seventh Annual Meeting of The Society of Thoracic Surgeons, New Orleans, Louisiana, United States of America (2001). Professor Enio Buffolo was honored for his pioneering contributions to off-pump coronary artery bypass surgery. Present at this memorable occasion were Sergio Almeida de Oliveira, José Wanderley Neto, Paulo Pego, Tomas Salerno, Domingo Braile, Enio Buffolo, Mário Oswaldo Vrandecic-Peredo, Ricardo Lima, Paulo Brofman, Fabio Jatene, José Teles, and Noedir Stolf (left to right).

In recognition of his pioneering work, the Academia Brasileira de Ciências honored Buffolo for his lifelong contributions to cardiovascular surgery, citing his research on OPCAB as one of the most influential achievements in Brazilian medical science. He was honored for his pioneering contributions to OPCAB surgery in the Thirty-seventh Annual Meeting of The Society of Thoracic Surgeons, New Orleans, Louisiana, United States of America (2001). Several leadings of Brazilian cardiac surgery were present (Figure 4).

## PERSONAL LIFE AND VALUES

**Fig. 5:**
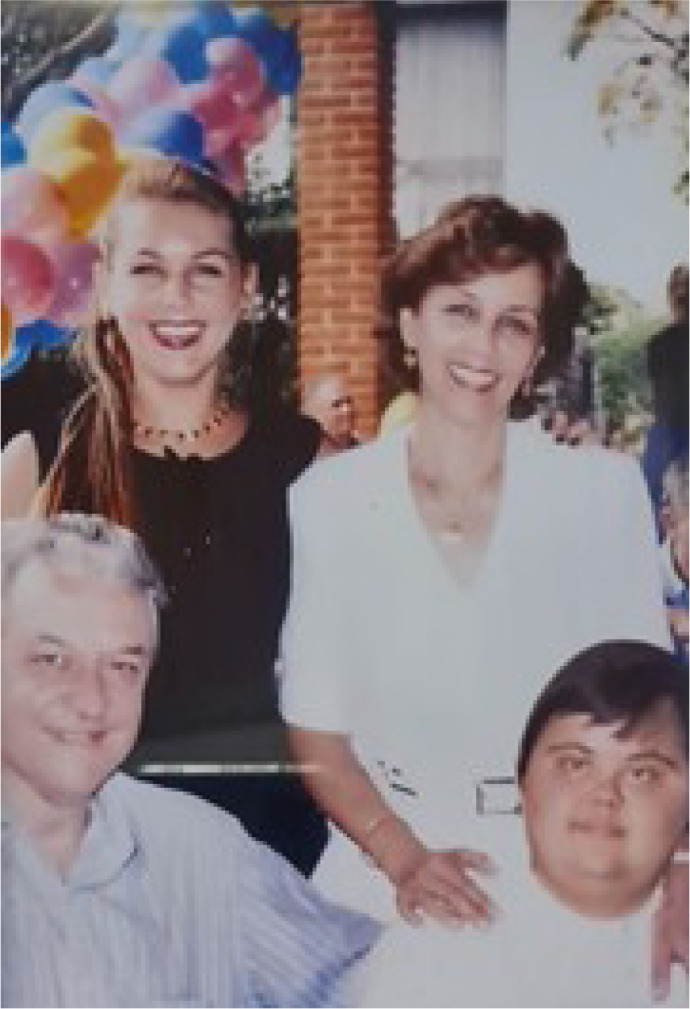
Enio Buffolo with his close family during a private moment. From left to right, Enio, Monica, Elza, and Marcelo.

**Fig. 6:**
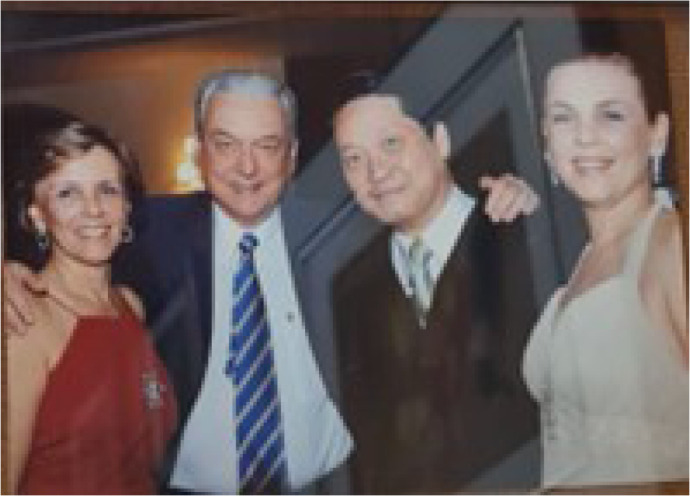
Enio Buffolo and his family during a gala dinner. From left to right, Elza, Enio, Sang, and Monica.

**Fig. 7 A and B:**
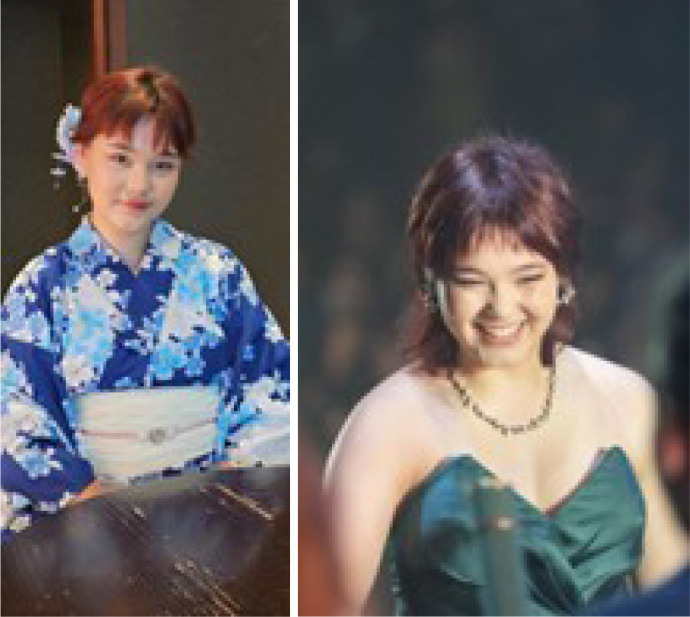
Enio and Elza Buffolo’s only and dearly loved granddaughter, blessed with a natural talent for drawing and painting.

**Fig. 8:**
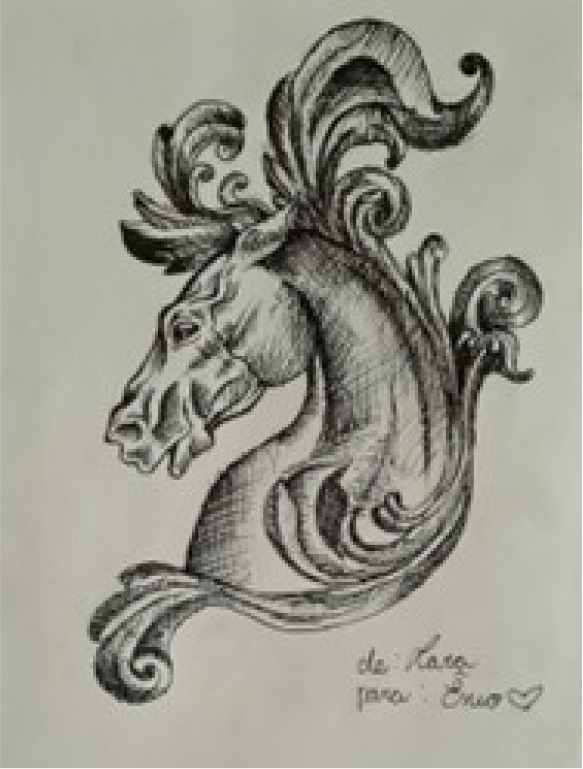
Gifted with exceptional artistic talent from young age, Lara created a heartfelt tribute to her grandfather, portraying through art the kindness and strength that defined his spirit, as well as his passion for horses following Doctor Sureño’s victory.

Buffolo married Elza Norma Zeigler the day after graduating from medical school. Their 60-year union was marked by deep companionship and mutual devotion. They had two children, Monica Zeigler Buffolo and Marcelo Zeigler Buffolo. Marcelo, who was born with Down Syndrome, is lovingly remembered for his warmth and resilience. Monica, a physician, is married to Dr. Sang Cha, MD, and their daughter, Lara Buffolo Cha, brought immense joy to her grandparents’ lives and displayed exceptional artistic talent from an early age (Figures 5, 6, 7A, 7B, and 8).

A man of faith and introspection, Buffolo’s spiritual journey led him from Catholicism to the practice of Vedanta philosophy, with daily meditation as a source of balance and insight. His humility, integrity, and quiet strength reflected the same discipline and compassion that defined his life and work, leaving a lasting impression on all who knew him.

## ACADEMIC LEGACY

Buffolo served on editorial boards of six journals, including The Annals of Thoracic Surgery and the Brazilian Journal of Cardiovascular Surgery. He received 39 academic awards and honors, authored 320 peer-reviewed publications with over 3,400 citations, wrote one book and 38 book chapters, supervised 46 master’s and PhD theses, participated in 48 academic committees, and co-held a patent for Brazil’s first endovascular prosthesis for aortic disease. Dr. Buffolo will be the posthumous recipient of the Life Achievement Award at the 11^th^ International Coronary Congress in New York on December 4^th^, 2025. Dr. Salerno will introduce Enio Buffolo and receive the tribute on his behalf, which will be sent to Elza Buffolo, Enio's wife.

## BRAZILIAN TURF

**Fig. 9:**
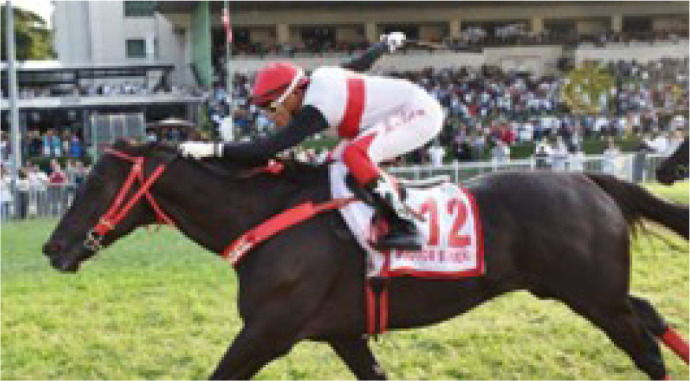
The horse Doctor Sureño, Enio Buffolo’s champion horse from Haras Moema, winner of the 100th São Paulo Grand Prix on May 7, 2023, at the Jockey Club of São Paulo, Brazil.

Passionate about racehorses, Dr. Enio, always partnering with his brother, Ernani Buffolo, through his Haras Moema, built a successful history on the racetrack in Brazil. The horse Doctor Sureño won the São Paulo Grand Prix (GP), Matias Machline GP, Latino Americano GP, and Vomage São Paulo GP; the horse Puerto-Madero won the Paulista Derby GP; and the horse Braço Forte won the Ipiranga GP and Brazilian Jockey Club GP; finally, the latter enabling Jeane Alves to become the first woman to win a top-ranked race at the Brazilian Jockey Club, and these are some of the great gifts his Haras Moema gave to Brazilian horse racing (Figure 9).

**Fig. 10:**
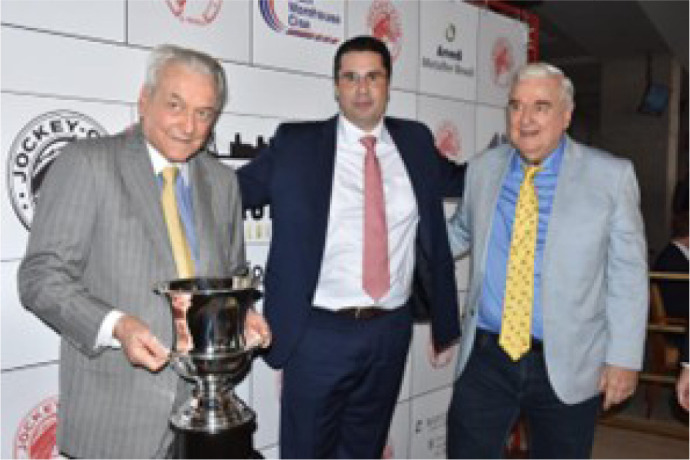
Enio Buffolo (left), his brother Ernane Buffolo (right), and at center, his nephew Fabricio Buffolo, a horse trainer in the United States of America, receiving the trophy of Doctor Sureño’s victory at the Jockey Club of São Paulo, Brazil, 2023.

Beyond being passionate about horse racing, he was always a model of respect, dedication, and dignity. Those fortunate enough to have had him as a friend or who received the attention and affection he always gave now have an irreparable loss, including the Turf Community and the Brazilian Jockey Club - SP (Figure 10).

## CONCLUSION: LEGACY AND IMPACT

Professor Enio Buffolo’s visionary leadership, academic excellence, and humanistic values transformed cardiovascular surgery in Brazil. His innovative spirit and ability to unite colleagues around shared goals created a generation of surgeons who carry forward his legacy. His passing marks the loss of a true pioneer - whose work changed lives, institutions, and the very practice of cardiac surgery. His contributions will remain deeply embedded in the history of Brazilian and global cardiovascular medicine.

Read more about Dr. Enio Buffo at: Curriculo Lattes/Plataforma Lattes

Address to access his CV: http://lattes.cnpq.br/9709188420552471

ID Lattes: 9709188420552471

Last updated on July 16^th^, 2020.

## Artificial Intelligence Usage

The authors declare use of ChatGPT solely to review English language grammar as necessary, and they take full responsibility for the content to be published.
